# The Resilience Scale in Portuguese Adults under Assisted Reproductive Techniques

**DOI:** 10.3390/ijerph19105932

**Published:** 2022-05-13

**Authors:** Joana Romeiro, Paulo Nogueira, Jenny Hall, Sílvia Caldeira

**Affiliations:** 1 Institute of Health Sciences, Universidade Católica Portuguesa, 1649-023 Lisbon, Portugal; scaldeira@ucp.pt; 2 Instituto de Medicina Preventiva e Saúde Pública, Faculdade de Medicina, Universidade de Lisboa, 1649-028 Lisboa, Portugal; paulonogueira@edu.ulisboa.pt; 3 Independent Researcher, Liverpool L17 7AQ, UK; drjennyhall@outlook.com

**Keywords:** adult, assisted reproductive techniques, factor analysis, infertility, instrument validation, resilience, psychological resilience

## Abstract

Assessing resilience response to an adverse event such as infertility requires measuring the same construct adequately and accurately by nurses. The objective of this study was to explore the validity and reliability of the Portuguese version of the Resilience Scale for adults. A cross-sectional and methodological design was used, and factor analyses were performed. The sample comprised 140 adult Portuguese individuals under fertility treatment recruited from health-related websites. The findings yielded a bad fit of the hypothesized Resilience Scale structure with the observed data. Instead, a 21-item tool with a four-factor structure revealed high internal consistency (0.94). The tool correlated positively and significantly to the Portuguese version of the Spiritual Well-Being Questionnaire and was negatively associated and lacked correlation with the Fertility Adjustment Scale. The 21-item Resilience Scale is a reliable tool suitable to measure resilience in Portuguese adults under assisted reproductive techniques. This tool offers the opportunity of early recognition by health professionals aiming to enhance patients’ coping skills effectively and promote positive psychological and mental health outcomes.

## 1. Introduction

Resilience is a critical concept in healthcare and has been studied in a wide diversity of contexts across the lifespan [1,2]. Different perspectives and definitions of resilience are easily found in the literature [3]. For instance, resilience has been grounded in the psychological domain, in neuroscientific and physiologic disciplines [3,4], and related to a socio-ecological view in terms of personal characteristics, outcomes, or processes [3,4]. Resilience has also been associated with the interaction between risk factors and inner strength [4,5]. On the other hand, resilience reflects a dynamic process that derives and is influenced by context and human responses to life events [6]. Resilience has also been described to have a fundamental role in the quality of life [7] and wellbeing [5,8]. A synthesis of concept analysis studies identified resilience as an “*ability to recover from perceived adverse or changing situations, through a dynamic process of adaptation, influenced by personal characteristics, family and social resources, and manifested by positive coping, control, and integration*” [9]. Often, empirical evidence focused on an individual’s adaptation to bereavement [8,10] and illness, in particular, in chronically ill populations and adolescents [2,4]. Research has found a link between high levels of resilience and increased adherence to treatment, predisposition to self-care, and healthier behaviors.

Additionally, such positive adaptation benefits were associated with low psychological and mental manifestations such as anxiety and depression [5,11,12,13,14].

Shiner and Masten (2012) and McGowan and collaborators (2018) claimed that resilience improved with age and with recurrent and repeated experiences in handling aversive life events. This process seems to help individuals face emerging challenges and develop coping strategies and resources potentially useful in the future [15]. Interestingly, regardless of the relation between time having the experience and resilience, when time is lived with a diagnosis, such as patients with HIV, lower levels of resilience may be identified [15]. The same researchers stated that more extended exposition to illness led individuals to experience prolonged stress, which affected the resources necessary for positive mental development [15]. Similar results were found in an early study conducted on women with infertility [16].

In clinical terms, infertility is a reproductive health condition resulting from a failure to have a clinical pregnancy in one year of regular unprotected sexual intercourse or the impaired ability of a person and/or a couple to reproduce [17].

Previous works identified that reactions to fertility problems followed a biopsychosocial model [18] and that they happened at the intrapersonal, interpersonal, and transpersonal levels simultaneously [19,20,21].

Just as resilience was described to function as a protective factor in a crisis [12], it also operated against the distress caused by infertility [22,23], but interestingly, also during pregnancy and puerperium [24]. Indeed, Li and collaborators [7] demonstrated that resilience played a determinant role between infertility-related stress and quality of living in a sample of 498 women [7]. Living longer with such a reproductive diagnosis reflected a declined ability to cope [16], and increased the levels of anxiety, depression, burden, and treatment withdrawal [25,26,27,28]. These effects were particularly relevant during assisted reproductive techniques (ARTs) [26,27,29]. Gameiro and collaborators (2016) predicted that one in every ten women living with such an infertility process had a compromised trajectory of adapting to stressors, which would be a cause for serious mental health impairment from 11 to 17 years after treatment.

Such undeniable and devastating consequences and responses are worth further research to raise awareness about human responses to infertility, conduct targeted individualized and person-centered interventions, and improve outcomes and quality in reproductive care.

Measurement tools have been developed to identify influencing factors in the perceived quality of living. Still, a thorough assessment of factors, like the case of a positive resource as resilience, depends on the accuracy and validity of instruments [3]. It is particularly evident concerning the Resilience Scale (RS) for adults that, despite receiving the best psychometric ratings by a methodological review of resilience measurement scales [3], it remains under-researched in adults, in a Portuguese population and in specific samples of individuals going through ART [30,31]. Hence, this study had the primary purpose of exploring the validity and reliability of the Portuguese version of the RS for adults. In addition, detailed discussions of the demographic and clinical characteristics of the sample and of the RS scores were performed.

## 2. Materials and Methods

### 2.1. Design and Participants

A descriptive cross-sectional and methodological design was conducted. A non-probabilistic and convenience sample of people with internet access was included. Inclusion criteria were participants who were: (1) adult men and women (aged 18 years or older); (2) Portuguese nationality; (3) in the process of engaging or during any stage of a fertility treatment; (4) willing to participate in this study; (5) agreed to and signed electronic informed consent. Incomplete questionnaires were excluded.

Recruitment took place through online invitations in fertility-related websites. This involved the release of periodical advertisements with potential participants asked to access a web-based questionnaire by clicking on an electronic link which directed them to the informed consent of the study.

The final sample of this research comprised 104 individuals.

### 2.2. Instrument

The online survey comprised demographic spiritual, religious, and clinical-health and treatment questions along with the Portuguese version of the RS for adults (previously translated and validated in a sample of 365 adults (students and church attendants) by Deep and Pereira (2012). This tool diverted from the original 25-item RS [2], which comprised five resilience factors: “Serenity”, “Perseverance”, “Self-confidence”, “The meaning of life”, and “Self-reliance”. The score of the original scale ranges from 25 to 175, and higher scores indicate higher degrees of resilience. The questions are answered on a Likert scale (from 1 to 7), which correspond to the lowest level of disagreement and the highest level of agreement [2]. Several studies have tested the original instrument in samples of caregivers of spouses with Alzheimer’s disease, graduate students, first-time mothers returning to work, residents in public housing, pregnant and postpartum women, and older adults. These results presented the acceptable internal consistency and reliability of the RS with a Cronbach’s alpha ranging between 0.76 and 0.91 [2].

Meanwhile, a remodeled version emerged with the same 25 original items and two subscales: “Personal Competence” (items 1, 2, 3, 4, 5, 6, 9, 10, 13, 14, 15, 17, 18, 19, 20, 23, and 24); and “Acceptance of Self and Life” (items 7, 8, 11, 12, 16, 21, 22, and 25) [2]. Nevertheless, the translated and validated Portuguese version [30] presented a shortened 23-item RS with four factors: “Perseverance” (known as Factor 1 including items 7, 12, 15, 16, 17, 21, and 22), “The meaning of life” (Factor 2 including items 3, 5, 8, 13, 14, and 19), “Serenity” (Factor 3 including items 6, 9, 10, 11, and 20) and “Self-reliance and self-confidence” (Factor 4 including items 1, 2, 4, 18, and 23) [28]. The total score in this adapted scale ranges between 25 and 161.

### 2.3. Data Collection

A web-based survey was designed and included information such as: demographic aspects; spiritual and religious beliefs; clinical-health data; and fertility treatments.

The length of time estimated for completion of the instrument was 10 to 15 min. The study adopted two phases. The first included a pilot study with previous review by three nursing experts. These experts were selected considering the following criteria: expertise on the method, expertise on the topic (spirituality or meaning), or expertise on the health condition. This was followed by a pretest with a sample of thirty respondents recruited by the same means. The pretest sample was composed mostly of females (97%) between 29 and 44 years old with a minimum of one year having an infertility diagnosis (17%). Most participants reported being married (60%) with higher degrees of study (47%) and employment (97%). The majority had no children of their own (93%) and reported the female factor as the main cause for their infertility (30%). Moreover, there was a predominance of persons waiting for the treatment to begin (57%). After the evaluation of the findings obtained in the prior test, along with suggestions made by the participants and experts, minor changes were made to the questionnaire as to provide clearer writing and understanding.

A final version of the survey was achieved and implemented in the second phase of the study. All subjects gave their informed consent for inclusion before they participated in the study.

The current study is part of the first author’s doctoral thesis.

### 2.4. Data Analysis

Statistical analyses were performed using SPSS, version 26.0 [32]. The 23 items of the RS were checked to confirm suitability in further analysis. The variables were screened using skewness and kurtosis, to gather information about the normal distribution of data [33]. To detect differences in the RS means by groups, the independent sample t-tests and the One-Way ANOVA (Table 1) were used following the Pestana and Gageiro (2003) premise of data normality in samples with 30 or more respondents. As a measure of construct reliability, the internal consistency was calculated using Cronbach’s α (recommended value higher than 0.70) [34]. Suitability to perform a factor analysis was determined with Bartlett’s Test of Sphericity (*p* < 0.05) [33] and the Kaiser–Meyer–Olkin (KMO) sampling adequacy test (higher than 0.6) [35]. A confirmatory factor analysis (CFA) using the AMOS SPSS program (Analysis of Moment Structures), version 26.0 [32] was previously conducted to test the four-factor structure and data fit of the 23-item RS. A good fit was achieved if goodness-of-fit indices presented the following recommended values: ratio of chi-square statistic to the respective degrees of freedom (*X*^2^/df) (lower than 3); the root mean square error of approximation (RMSEA) with a value of 0.01 (excellent fit), below 0.05 (good fit), lower than 0.08 (good fit), between 0.05 and 0.10 (moderate fit), and higher than 0.10 (bad fit); the incremental fit index (CFI) (greater than 0.90–0.95); Tucker–Lewis index (TLI) ranging between 0 and 1 with values greater than 0.90 indicating good fit; and Normed Fit Index (NFI) higher than 0.90 [36,37,38]. If a bad fit statistic was noted, in further examination of the Deep and Pereira (2012) model, an exploratory factor analysis (EFA) would be conducted instead. Like Deep and Pereira (2012) and Wagnild and Young (1993), a Varimax rotation was performed. Items with communalities less than 0.2 were removed during this process. A principal component extraction was performed based on eigenvalues greater than one (Kaiser criterion or K1) [39]. Following previous RS studies, only factors with an explained variance above 5% were retained. Moreover, factors with at least three items and with a loading greater than 0.4 and low cross-loading were retained. The absolute values of factor loadings less than 0.3 were suppressed. Additional scree plot analysis was used to compare findings. After the solution stabilized, the total variance explained by the retained factors was at least 50% or higher, and acceptable correlations between factors were checked.

Lastly, the convergent and divergent validity of the final RS structure was further investigated using Pearson’s correlation coefficients between each variable of the RS and the Portuguese version of the Spiritual Well-Being Questionnaire (SWBQp) [40] and the Fertility Adjustment Scale (FAS) [41,42].

The SWBQp is a well-established tool for self-assessment of spiritual wellbeing in the Portuguese population [40]. It consists of 20 items and four subscales: personal (items 5, 9, 14, 16, and 18), communal (items 1, 3, 8, 17, and 19), environmental (items 4, 7, 10, 12, and 20), and transcendental (items 2, 6, 11, 13, and 15) [43]. This instrument and its construct fall within the spiritual domain, and the instrument was selected because of its hypothesized relationship with the resilience construct [5,8]. The FAS is a tool intended to measure specific psychological reactions to fertility problems and treatment outcomes [44]. It comprises 10 items and three subscales: “Centrality of parenting” (1, 3, 6, and 9); “Suspended life” (items 2, 5, and 8); “Acceptation of life without children” (Items 4, 7, and 10) [41,42]. The reliability of the RS was assessed using Cronbach’s alpha of the total scale and subscales (>0.7) [34].

## 3. Results

### 3.1. Sample Characteristics

One hundred and four participants were integrated in the sample of this study since there were no missing or incomplete answers. The sample included 102 women (98.1%) and 2 men (1.9%); their age ranged from 26 to 54 years with a mean of 35.4 (SD = 4.8; 95% CI = 34.49–36.34); the majority lived in the north region of Portugal (33.3%), were married (57.7%), and living together for a mean of 7.97 years (SD = 4.80; 95% CI = 7.03–8.91); most had a higher education level (65.4%), were employed (87.5%), and were intellectual and scientific experts (35.6%). The primary form of infertility predominated in the sample (86.5%). About 47.1% of the respondents were on the eminence of starting a new medical fertility procedure. In Vitro Fertilization (IVF) was the most common ART procedure (52.6%) (Table 1).

### 3.2. Descriptive Analysis of RS

A significant association was found between resilience and: spiritual changes with diagnosis (*p* = 0.009); spiritual changes with treatment (*p* = 0.005); religion changes with treatment (*p* = 0.010); and cause of infertility (*p* = 0.014) (Table 1). Resilience was more relevant in people that reported: no change in their spirituality when diagnosed with infertility (M = 5.24; SD = 0.86) or during fertility treatment (M = 5.27; SD = 0.83); no change in religion during treatment (M = 5.24; SD = 0.87); both a female and male cause (M = 5.21; SD = 0.82). The highest mean scores of the scale were presented by plant and machine operators (M = 5.82; SD = 0.00); students (M = 5.54; SD = 0.03); people undergoing another type of fertility treatment (M = 5.50; SD = 1.13); and people who perceived spirituality as very important to them (M = 5.43; SD = 0.99) (Table 1).

The mean item scores ranged from 3.97 (SD = 1.97) (item 9) to 5.61 (SD = 1.47) (item 16) with a mean of items for the overall scale of 4.96 (SD = 1.05) (Table 2). The total score for the RS was 114.12 (SD = 24.25).

The Cronbach’s alpha indicated the high internal consistency or homogeneity of the RS (0.94) (Table 2). Before testing the factor structure of the RS [44], its appropriateness was confirmed to proceed with the Bartlett’s Test of Sphericity ((χ^2^ = 1619.36, df = 253, *p* < 0.001) and with a KMO of 0.90).

### 3.3. Exploratory Factor Analysis (EFA)

The Kaiser-Guttman criterion (K1) (Table 3) (eigenvalue > 1) was used, and a Varimax rotation presented an initial scale structure with five factors (Table 4). Yet, a factor with two variables is only considered reliable when the correlation between variables is high (r > 0.70), and this did not happen between variables 10 and 11 in Factor 5 (*r* = 0.48) (Table 4). Following the procedure of the original scale and Deep and Pereira’s (2012) version, only three factors with an explained variance above 5% were retained, which explained 60.05% of the total variance (Table 3).

Additionally, communalities after extraction ranged between 0.29 and 0.80 (Table 5). As such, item 18 was eliminated since recommended values should be above 0.30.

**Figure 1:**
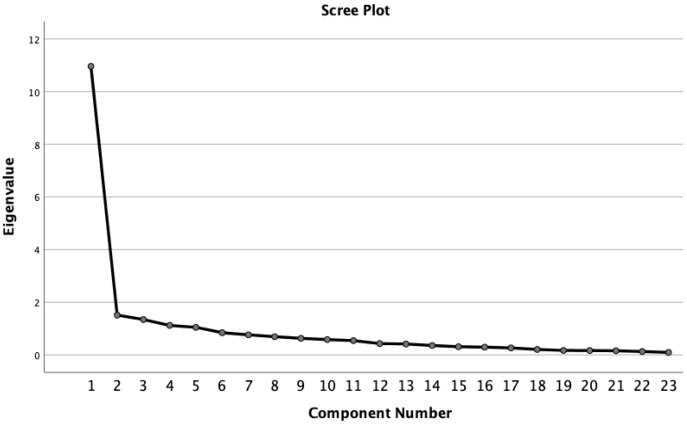
Scree plot representing the eigenvalues (23-item Resilience Scale).

The analyzed structure had satisfactory loadings of each item on their respective factors (>0.30) (Table 4). No cross-loadings were observed. These results, along with the scree plot (Figure 1), seemed to propose a four- or three-factor solution.

These solutions were further investigated when removing item 10 due to low pattern coefficients (<0.40) (Table 4) and led to the further analysis of the 21-item structure of the RS. The assessment of the KMO (0.90) and Bartlett’s Test of Sphericity (χ^2^ = 1514.725, df = 210, *p* < 0.001) allowed us to proceed with an EFA. A PCA with Varimax rotation and scree plot analysis presented a four-factor solution with a cumulative explained variance of 68.17%, which was higher than the percentage obtained with the previously analyzed structures (Table 3). At this stage, the 21-item scale included Factor 1 (items 5, 6, 12, 13, 14, and 19), Factor 2 (items 1, 2, 3, 4, and 11), Factor 3 (items 7, 8, 15, 16, 17, and 22), and Factor 4 (items 9, 20, 21, and 23) (Table 4). Factor 1 was labeled “The meaning of life”, Factor 2 “Self-reliance”, Factor 3 “Self-confidence”, and Factor 4 “Acceptance of Life circumstances”.

Internal consistency of the overall 21-item scale (0.95) would not significantly improve with the deletion of any observed variable, and as such no other item was deleted (Table 6).

**Figure 2:**
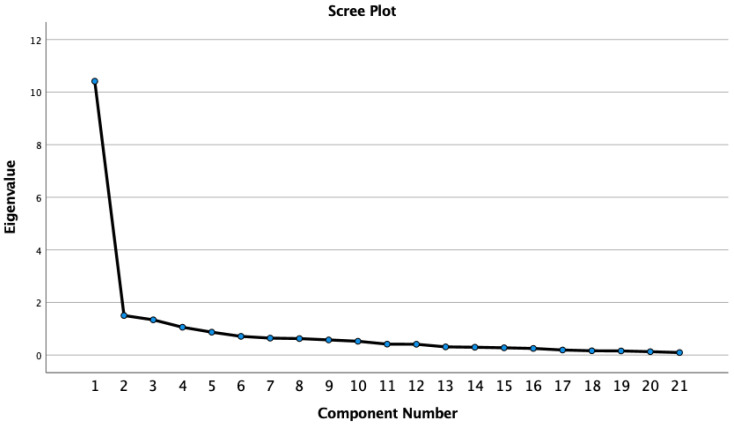
Scree plot representing the eigenvalues (21-item Resilience Scale).

Increased pattern coefficients (>0.40) and higher communalities were shown in the new solution (Table 5). Factor loadings ranged from 0.44 to 0.82 (Table 4), and the scree plot (Figure 2) confirmed the four-factor structure of the 21-item RS. Skewness and kurtosis values for these factors did not exceed the critical values of 1 and 2, respectively, which means that the normality was met for this sample (Table 6).

The correlations between Factor 1, Factor 2, Factor 3, and Factor 4 were significant, ranging between 0.538 and 0.799 (Pearson coefficient (*r*) ≥ 0.30) (Table 7).

On the other hand, the 21-item scale presented an overall mean of 104.06 (SD = 22.68) and an item’s mean of 4.95 (SD = 1.08).

### 3.4. Reliability

The final shortened RS version presented good reliability (0.94) as well as for all the subscales (0.76–0.90) (Table 6).

### 3.5. Convergent and Discriminant Validity

The convergent validity of the 21-item RS was further investigated by comparing it to the SWBQp and the FAS. Spiritual wellbeing and resilience were theoretically related, and the hypotheses of a strong and positive correlation between the two constructs was confirmed in this study (*r* = 0.412; *p* < 0.001) (Table 8).

On the other hand, correlations between RS and FAS indicated discriminant validity (*r* = −0.026; *p* = 0.790), with the majority of Pearson’s values indicating the uniqueness of the four RS factors with the exception of the fourth RS factor, which had a significant correlation with all FAS domains (r ranged between −0.249 and 0.290; *p* < 0.05). Finally, the last factor of the FAS (“Acceptation of life without children”) had a significant correlation with the overall RS (*r* = 0.227; *p* = 0.021) and with each of its subscales (Table 8).

## 4. Discussion

The aim of the present study was attained. It evaluated the psychometric properties of the Portuguese version of the RS [30] in a sample of adults going through fertility treatment. No other study has been found that has tested the factor structure in such a sample and environment. Indeed, the RS has been used in younger participants [45,46,47,48,49,50,51] although it was developed using older women [2,3]. Moreover, measurement instruments related to resilience are under-researched in settings such as ART [9,20,21,52], and this study expresses the utility of the RS in reproductive care as it facilitates the assessment of the impact of infertility and ART on being resilient and coping with such adversity.

The findings of the CFA yielded a bad fit of the hypothesized RS structure with the observed data. Instead, an EFA of the RS was conducted and revealed four factors (Factor 1 “The meaning of life”, Factor 2 “Self-reliance”, Factor 3 “Self-confidence”, and Factor 4 “Acceptance of Life Circumstances”). The number of extracted components resembled Deep and Pereira’s (2012) four-factor version, although with a shorter 21-item structure and a different item distribution. Therefore, the results of this study deviated from the original 25-item as earlier presented by Wagnild and Young (1993).

The 21-item RS indicated high internal consistency (0.94) and good reliability for all four subscales (0.76–0.90), higher than those obtained by Deep and Pereira (2012) but with similarities to Wagnild and Young’s (1993) study (0.76–0.91).

There were strong intercorrelations between all four factors of the RS. Additionally, the RS was positively and significantly related to SWBQp, but such a relationship is slightly limited between Factor 2 of the RS and the communal, environmental, and transcendental SWBQp subscales. Together, these results suggest that the two measurement tools evaluate different constructs yet with great resemblance. These are not surprising results, as they confirm the already-addressed association between resilience and spiritual wellbeing and the tendency of resilient individuals to manifest a higher sense of wellbeing [5,8]. Furthermore, spiritual wellbeing has been highlighted as a strong predictor of mental health [53].

On the other hand, the RS was negatively associated and lacked correlation to the FAS. Therefore, the results suggest that these tools evaluate different constructs which are structurally independent. Despite these findings, the last dimension of the FAS correlated positively to all the subscales of the RS. These findings were already expected as “Acceptation of life without children” embodies to a certain degree the resilience definition [9], because it relates to an existing adaptation to an adverse life event, in this case, adaptation to involuntary childlessness.

Meanwhile, a slightly high resilience level was found in the sample, which confirms an adaptive process emerged and prevailed in individuals facing infertility [2,10], and acted as a protective factor against such event [23].

Nevertheless, it must be recalled that most of the participants in our sample were at the initial stages of ART and waiting for it to begin, which might evoke increased levels of positive expectation in a successful pregnancy. In fact, the previous literature addressed the frequent emotional ride lived by individuals characterized by hope at the beginning of a cycle [20,21], and a study exploring how personality traits are related to resilience development indicated hope was a significant predictor of resilience [5]. Moreover, the positive association between resilience and engagement in action-focused coping behaviors [18] could also explain findings of increased resilience in individuals pursuing ART.

This study also pointed to a significant association between resilience and spirituality during infertility diagnosis and spirituality/religion during treatment. Both spirituality and religion have been recognized as sources of resilience and as means of supporting individuals in their path to recover or deal with a disease, mitigate the effects of such adversity and help regain and/or maintain health [54,55]. A correlation between spirituality and resilience has been highlighted before, with reports of a positive and dynamic link that occasioned the use of the term “spiritual resilience” [55].

Additionally, a significant association was also found between resilience and cause of infertility, with a higher resilience score when a female and male factor were responsible for couples’ infertility. Often, women have perceived themselves as responsible for raising a family and being accountable for becoming pregnant [56]. As such, a shared responsibility between partners in the cause of couples’ infertility might justify decreased levels of stress in women and higher levels of resilience in our sample, as most respondents were females.

Even though findings from this study add to the growing body of evidence, some limitations must be mentioned. First, the RS is a tool that was not specifically designed to assess peculiarities related to living with infertility and specifically designed to be applied in a sample under ART. Plus, women participated more than men, and having a convenience sample recruited through the web might have compromised the generalization of the results to the wider population due to its voluntary nature.

## 5. Conclusions

Data showed the 21-item RS is a valid and reliable tool in this sample. Although aspects related to the sampling method and criteria were rigorous, it might be difficult to generalize the findings to the broader population of Portuguese individuals under fertility treatment. Moreover, it must be remembered that resilience has a subjective nature, and it is difficult to ascertain if different patients will respond to the same phenomenon in the same way as the sample. In addition, a predominance of women and scarcity of male participants might have introduced some bias, as resilience development and coping strategies towards infertility are known to happen in different ways according to gender [57]. The aforementioned aspects alert us to the need for further research in men and couples. It is crucial to understand how resilience evolves between partners and affect marital living and quality of life during different stages of ART. Since resilience was found to be affected by age, it would be interesting to explore how resilience evolves in time with ART and the increase in women’s age in treatment, and how personality traits influence adaptation to infertility and treatment. Comparison with other findings is crucial, and future research is advised to follow similar methodological procedures which would help prove the scientific robustness of the tool.

This work offers a new insight towards the measure of resilience and the potential of the RS to be used to increase desired patient outcomes in reproductive healthcare. Specific interventions of practitioners based on such assessments could be useful in clinical practice to help patients become more resilient when dealing with infertility and ART. Effective skills to cope with infertility enhance resilient behavior, prevent negative, devastating, and long-term effects in general and mental health, and positively impact the quality of life and wellbeing of individuals living in such circumstances.

This study demonstrates the crucial role researchers, practitioners, and policymakers have in measuring resilience, as it may provide more accurate health projections and the design of specific strategies towards the provision of a more patient-oriented and integrated care. Developing resilience-based programs to address and support patients with infertility may help enhance long-term resilience, facilitate self-confidence, self-efficacy, life transition, and prevent fertility treatment withdrawals.

## Figures and Tables

**Table 1:** Mean scores of the RS by characteristics of participants (*n* = 104).

			Resilience Scale (RS)		
Variable			Response Mean (SD)	*p*	Total Mean (SD)
**Social—Demographic**					
**Gender**				0.248 ^a^	
		Female	4.94 (1.05)		113.81 (24.35)
		Male	5.65 (0.84)		130.00 (11.31)
**Age**				0.807 ^b^	
		≤34	4.78 (1.08)		110.08 (25.06)
		35–40	5.09 (1.01)		117.21 (23.25)
		41–51	5.03 (1.11)		115.90 (25.53)
		≥52	5.69 (0.00)		131.00 (0.00)
**Marital Status**				0.360 ^b^	
		Married	4.94 (1.09)		113.83 (25.21)
		Together	4.97 (1.03)		114.44 (23.70)
		Divorced/Separated	4.75 (1.34)		109.33 (30.85)
		Single	5.24 (0.35)		120.66 (8.08)
**Current relationship (years)**				0.359 ^b^	
		≤3	4.72 (1.17)		108.66 (27.01)
		4–6	5.14 (1.16)		118.33 (26.76)
		7–9	4.80 (0.84)		110.42 (19.47)
		≥10	5.05 (1.08)		116.27 (24.91)
**Education level**				0.509 ^b^	
		Middle school	5.95 (0.00)		137.00 (0.00)
		High school	4.66 (1.10)		107.19 (25.31)
		Professional course	5.38 (0.90)		123.77 (20.83)
		Bachelor/Graduation	4.92 (1.07)		114.12 (24.82)
		Master	5.06 (1.02)		116.41 (23.49)
		PhD	5.42 (0.45)		124.66 (10.40)
**Employment status**				0.053 ^b^	
		Employed	5.00 (1.02)		115.01 (23.52)
		Unemployed	4.53 (1.32)		104.36 (30.46)
		Student	5.54 (0.03)		127.50 (0.70)
**Occupation**				0.806 ^b^	
		Representatives of the legislative branch of executive bodies, officers, directors, and executive managers	5.37 (1.46)		123.60 (33.65)
		Experts from intellectual and scientific activities	5.11 (0.97)		117.62 (22.46)
		Intermediate level technicians and professions	4.15 (1.33)		95.50 (30.68)
		Administrative staff	5.06 (1.01)		116.57 (23.36)
		Personal service, security, and safety workers and salespeople	4.79 (0.86)		110.22 (19.81)
		Skilled workers in industry, construction, and craftsmen	3.91 (0.00)		90.00 (0.00)
		Plant and machine operators	5.82 (0.00)		134.00 (0.00)
**Spirituality—Religion**					
**Spiritual person**				0.659 ^a^	
		No	4.90 (1.06)		112.92 (24.40)
		Yes	4.97 (1.05)		114.50 (24.34)
**Spiritual importance**				0.940 ^b^	
		Not important	4.98 (1.03)		114.71 (23.85)
		Little important	4.74 (1.09)		109.08 (25.12)
		Important	4.93 (1.04)		113.42 (23.98)
		Very important	5.43 (0.99)		124.93 (22.91)
**Spiritual changes with diagnosis**				0.009 ^b^	
		No change	5.24 (0.86)		120.71 (19.94)
		Less important	4.43 (1.18)		102.05 (27.33)
		More important	4.79 (1.13)		110.20 (26.02)
**Spiritual changes with treatment**				0.005 ^b^	
		No change	5.27 (0.83)		121.30 (19.25)
		Less important	4.79 (1.31)		110.26 (30.27)
		More important	4.57 (1.10)		105.16 (25.40)
**Religious person**				0.888 ^a^	
		No	4.87 (1.02)		112.14 (23.64)
		Yes	5.00 (1.07)		115.08 (24.64)
**Importance of religion**				0.701 ^b^	
		Not important	5.16 (1.05)		118.72 (24.33)
		Little important	4.74 (1.16)		109.04 (26.85)
		Important	4.98 (1.00)		114.66 (23.16)
		Very important	5.16 (1.19)		118.71 (27.42)
**Religion changes with diagnosis**				0.239 ^b^	
		No change	5.15 (0.96)		118.61 (22.14)
		Less important	4.90 (1.21)		112.82 (27.96)
		More Important	4.66 (0.99)		107.33 (22.89)
**Religion changes with treatment**				0.010 ^b^	
		No change	5.24 (0.87)		120.54 (20.09)
		Less important	4.91 (1.34)		113.08 (30.83)
		More important	4.58 (0.97)		105.51 (22.49)
**Clinical—Infertility**					
**Type**				0.090 ^a^	
		Primary	4.99 (1.02)		114.96 (23.55)
		Secondary	4.72 (1.24)		108.71 (28.69)
**Nature**				0.128 ^b^	
		Never been pregnant	4.94 (0.99)		113.84 (22.86)
		Natural pregnancy without live birth	5.15 (1.10)		118.55 (25.46)
		Natural pregnancy, had child, not able to have another child	4.55 (1.44)		104.77 (33.27)
		Pregnancy with treatment, did not have a child	5.00 (1.09)		115.00 (25.18)
		Pregnancy with treatment, had child, not able to have another child	5.03 (0.83)		115.80 (19.11)
**Cause**				0.014 ^b^	
		Female	5.05 (0.90)		116.36 (20.88)
		Male	4.90 (1.34)		112.80 (30.96)
		Mixed	5.21 (0.82)		119.83 (19.02)
		Unknown	4.73 (1.10)		108.94 (25.40)
		Waiting for a diagnosis	4.53 (1.46)		104.33 (33.67)
**Diagnosis (years)**				0.980 ^b^	
		≤3	5.00 (1.08)		115.10 (24.98)
		4–6	4.95 (1.02)		113.85 (23.55)
		7–9	4.91 (1.05)		113.00 (24.26)
		≥10	4.63 (1.05)		106.71 (24.87)
**Consultation (years)**				0.185 ^b^	
		≤3	5.04 (1.05)		116.04 (24.26)
		4–6	4.83 (0.93)		111.21 (21.48)
		7–9	4.81 (0.97)		110.70 (22.46)
		≥10	4.51 (1.64)		103.80 (37.91)
**Treatments**					
**Previous treatments**				0.923 ^a^	
		No	5.11 (1.06)		117.70 (24.42)
		Yes	4.83 (1.03)		111.17 (23.90)
**Time in current treatment (months)**					
		≤3	4.21 (1.54)		97.00 (35.53)
		4–6	4.25 (1.16)		97.75 (26.84)
		7–12	4.91 (1.07)		112.93 (24.79)
		13–24	5.23 (0.91)		120.46 (21.00)
		24–36	4.76 (1.06)		109.60 (24.60)
		≥37	4.71 (0.98)		108.37 (22.71)
**Current treatment**				0.343 ^b^	
		Previous tests	4.87 (1.24)		112.15 (28.60)
		Waiting to start	4.98 (0.96)		114.65 (22.22)
		In cycle	4.84 (0.98)		111.47 (22.67)
		OI	5.30 (0.00)	0.589 ^b^	122.00 (0.00)
		IUI	4.47 (1.78)		103.00 (41.01)
		IVF	4.72 (0.97)		108.70 (22.51)
		ICSI	4.89 (0.98)		112.50 (22.63)
		Other	5.50 (1.13)		126.50 (26.16)
		Tests after cycle	5.13 (1.05)		118.12 (27.75)

Legend: ^a^ = Independent sample T —Student’s Test (Levene’s Test); ^b^ = One Way ANOVA test; SD = Standard Deviation; OI—Ovulation Induction; IUI—Intrauterine Insemination; IVF—In Vitro Fertilization; ICSI—Intracytoplasmic Sperm Injection.

**Table 2:** Descriptive statistics, Cronbach’s alpha coefficient of RS, and EFA assessment of normality of items.

Item	Mean	SD	Skewness	SE	Kurtosis	SE	Cronbach’s Alpha (without Item)
**1.**	5.43	1.41	−0.74	0.23	−0.45	0.46	(0.94)
**2.**	5.17	1.41	−0.60	0.23	−0.38	0.46	(0.94)
**3.**	5.28	1.43	−0.78	0.23	−0.02	0.46	(0.94)
**4.**	5.02	1.49	−0.60	0.23	−0.25	0.46	(0.94)
**5.**	5.17	1.62	−0.71	0.23	−0.31	0.46	(0.94)
**6.**	5.00	1.65	−0.72	0.23	−0.28	0.46	(0.94)
**7.**	5.25	1.53	−1.01	0.23	0.32	0.46	(0.94)
**8.**	5.32	1.50	−0.80	0.23	−0.13	0.46	(0.94)
**9.**	3.97	1.87	−0.09	0.23	−1.15	0.46	(0.94)
**10.**	5.10	1.46	−0.62	0.23	0.01	0.46	(0.94)
**11.**	4.68	1.63	−0.40	0.23	−0.60	0.46	(0.94)
**12.**	4.62	1.49	−0.28	0.23	−1.00	0.46	(0.94)
**13.**	4.81	1.50	−0.43	0.23	−0.76	0.46	(0.94)
**14.**	4.88	1.49	−0.46	0.23	−0.81	0.46	(0.94)
**15.**	4.95	1.53	−0.49	0.23	−0.73	0.46	(0.94)
**16.**	5.61	1.47	−1.13	0.23	0.79	0.46	(0.94)
**17.**	5.23	1.43	−0.75	0.23	−0.04	0.46	(0.94)
**18.**	4.97	1.47	−0.54	0.23	−0.43	0.46	(0.94)
**19.**	5.06	1.61	−0.47	0.23	−0.73	0.46	(0.94)
**20.**	4.33	1.62	−0.17	0.23	−0.69	0.46	(0.94)
**21.**	4.86	1.41	−0.52	0.23	−0.49	0.46	(0.94)
**22.**	4.58	1.63	−0.25	0.23	−0.86	0.46	(0.94)
**23.**	4.85	1.81	−0.60	0.23	−0.74	0.46	(0.94)
**Total**	**4.96**	**1.05**	**−0.53**	**0.23**	**−0.54**	**0.46**	**0.94**

CI = Confidence Interval; SD = Standard Deviation; SE = Standard Error.

**Table 3:** Components’ extraction from data based on K1 criterion and total variance explained for RS factor structure.

Components’ Extraction Based on K1 Criterion and Percentage (%) of Variance									
Component	23 Items			22 Items			21 Items		
	Total	% Variance	Cumulative %	Total	% Variance	Cumulative %	Total	% Variance	Cumulative %
1	10.96	47.65	47.65	10.41	47.34	47.34	9.79	48.99	48.99
2	1.51	6.56	54.21	1.50	6.86	54.20	1.48	7.41	56.41
3	1.34	5.83	60.05	1.35	6.14	60.34	1.34	6.70	63.11
4				1.13	5.14	65.49	1.011	5.05	68.17

**Table 4:** Value of Varimax rotation factor loading ^a^.

Varimax Rotation Factor Loading																							
Item	Five-Factor (23 Items)					Four-Factor (23 Items)				Three-Factor (23 Items)			Four-Factor (22 Items)				Three-Factor (21 Items)			Four-Factor (21 Items)			
	1	2	3	4	5	1	2	3	4	1	2	3	1	2	3	4	1	2	3	1	2	3	4
1.		0.65					0.67				0.69			0.69			0.72				0.69		
2.		0.80					0.80				0.82			0.82			0.83				0.82		
3.		0.66					0.54				0.58			0.57			0.63				0.62		
4.		0.75					0.75				0.76			0.76			0.75				0.78		
5.	0.74					0.75				0.72			0.72						0.63	0.73			
6.	0.57					0.59				0.60			0.61				0.53			0.59			
7.			0.56				0.54				0.60			0.60			0.64					0.56	
8.			0.52				0.54				0.59			0.59			0.65					0.54	
9.				0.65					0.63	0.55			0.56						0.53				0.67
10.					0.84				0.72			0.36				0.92							
11.					0.66				0.55		0.44			0.46			0.45				0.44		
12.	0.62					0.64				0.64			0.65						0.58	0.64			
13.	0.79					0.79				0.81			0.82						0.74	0.81			
14.	0.73					0.74				0.76			0.76						0.69	0.74			
15.			0.51			0.47					0.49			0.49			0.55					0.53	
16.			0.80					0.76				0.66			0.67			0.69				0.80	
17.			0.68					0.65				0.62			0.62			0.63				0.70	
18.		0.34							0.36		0.36												
19.	0.67					0.69				0.69			0.69						0.67	0.66			
20.				0.81				0.68				0.78			0.79			0.80					0.81
21.				0.53				0.63				0.66			0.66			0.68					0.53
22.			0.52					0.62				0.60			0.61			0.65				0.50	
23.				0.51				0.48				0.58			0.57			0.59					0.53

^a^ For clarity, pattern coefficients < 0.3 are not shown.

**Table 5:** Communalities.

Communalities			
		23-Items	21-Items
Item	Initial	Extraction	Extraction
1. I usually manage one way or another	1.000	0.57	0.59
2. I am able to depend on myself more than anyone else	1.000	0.73	0.76
3. Keeping interested in things is important to me	1.000	0.56	0.64
4. I can be on my own if I have to	1.000	0.64	0.67
5. I feel proud that I have accomplished things in life	1.000	0.74	0.74
6. I am friends with myself	1.000	0.67	0.67
7. I feel that I can handle many things at a time	1.000	0.69	0.74
8. I am determined	1.000	0.66	0.74
9. I seldom wonder what the point of it all is	1.000	0.46	0.72
10. I take things one day at a time	1.000	0.30	-
11. I can get through difficult times because I’ve experienced difficulty before	1.000	0.35	0.34
12. I have self-discipline	1.000	0.63	0.67
13. I keep interested in things	1.000	0.80	0.80
14. I can usually find something to laugh about	1.000	0.71	0.72
15. My belief in myself gets me through hard times	1.000	0.66	0.69
16. In an emergency, I’m someone people can generally rely on	1.000	0.53	0.73
17. I can usually look at a situation in a number of ways	1.000	0.70	0.75
18. Sometimes I make myself do things whether I want to or not	1.000	0.29	-
19. My life has meaning	1.000	0.59	0.61
20. I do not dwell on things that I can’t do anything about	1.000	0.62	0.75
21. When I’m in a difficult situation, I can usually find my way out of it	1.000	0.64	0.66
22. I have enough energy to do what I have to do	1.000	0.66	0.68
23. It’s okay if there are people who don’t like me	1.000	0.51	0.54

**Table 6:** Descriptive statistics, Cronbach’s alpha coefficient of RS models, and EFA assessment of normality of factors.

MIST Structure	Factor	Mean	SD	Skewness	Std. Error	Kurtosis	Std. Error	Cronbach’s Alpha
**23-item** **5-factor**		**4.96**	**1.05**	**−0.531**	**0.237**	**−0.547**	**0.469**	**0.940**
	Factor 1	4.92	1.28	−0.585	0.237	−0.470	0.469	0.905
	Factor 2	5.17	1.08	−0.522	0.237	−0.522	0.469	0.809
	Factor 3	5.15	1.25	−0.796	0.237	−0.796	0.469	0.904
	Factor 4	4.50	1.28	−0.191	0.237	−0.191	0.469	0.761
	Factor 5	4.88	1.33	−0.400	0.237	−0.400	0.469	0.646
**23-item** **4-factor** **[44]**		**4.96**	**1.05**	**−0.531**	**0.237**	**−0.547**	**0.469**	**0.940**
	Factor 1	5.01	1.21	−0.547	0.237	−0.432	0.469	0.909
	Factor 2	5.08	1.22	−0.666	0.237	−0.353	0.469	0.887
	Factor 3	4.61	1.16	−0.234	0.237	−0.750	0.469	0.741
	Factor 4	5.08	1.10	−0.587	0.237	−0.457	0.469	0.775
**23-item** **4-factor**		**4.96**	**1.05**	**−0.531**	**0.237**	**−0.547**	**0.469**	**0.940**
	Factor 1	4.92	1.26	−0.525	0.237	−0.686	0.469	0.915
	Factor 2	5.24	1.15	−0.628	0.237	−0.465	0.469	0.876
	Factor 3	4.90	1.21	−0.411	0.237	−0.605	0.469	0.864
	Factor 4	4.68	1.13	−0.227	0.237	−0.859	0.469	0.657
**23-item** **3-factor**		**4.96**	**1.05**	**−0.531**	**0.237**	**−0.547**	**0.469**	**0.940**
	Factor 1	4.78	1.26	−0.438	0.237	−0.665	0.469	0.895
	Factor 2	5.11	1.08	−0.565	0.237	−0.435	0.469	0.888
	Factor 3	4.93	1.14	−0.415	0.237	−0.691	0.469	0.858
**22-item** **4-factor**		**4.96**	**1.06**	**−0.534**	**0.237**	**−0.592**	**0.469**	**0.947**
	Factor 1	4.78	1.26	−0.438	0.237	−0.665	0.469	0.895
	Factor 2	5.13	1.12	−0.567	0.237	−0.512	0.469	0.889
	Factor 3	4.90	1.21	−0.411	0.237	−0.605	0.469	0.864
	Factor 4	5.09	1.46	−0.623	0.237	0.016	0.469	--
**21-item** **3-factor**		**4.95**	**1.08**	**−0.526**	**0.237**	**−0.599**	**0.469**	**0.946**
	Factor 1	5.12	1.13	−0.678	0.237	−0.437	0.469	0.903
	Factor 2	4.90	1.21	−0.411	0.237	−0.605	0.469	0.864
	Factor 3	4.75	1.25	−0.391	0.237	−0.826	0.469	0.873
**21-item** **4-factor**		**4.95**	**1.08**	**−0.526**	**0.237**	**−0.599**	**0.469**	**0.946**
	Factor 1	4.90	1.22	−0.569	0.237	−0.502	0.469	0.905
	Factor 2	5.11	1.11	−0.362	0.237	−0.576	0.469	0.810
	Factor 3	5.15	1.25	−0.796	0.237	0.146	0.469	0.904
	Factor 4	4.50	1.28	−0.191	0.237	−1.029	0.469	0.761

**Table 7:** Pearson correlations between extracted factors (*n* = 104).

Pearson Correlation between Extracted Factors				
Factor	1	2	3	4
1	1			
2	0.682 **	1		
3	0.799 **	0.704 **	1	
4	0.642 **	0.538 **	0.675 **	1

** Correlation is significant at the 0.01 level (2-tailed).

**Table 8:** Discriminant validity of the reconfigured Resilience Scale factors using SWBQp and FAS variables.

	Discriminant Validity of the Reconfigured Resilience Scale Factors									
	RS Total		RS—Factor 1		RS—Factor 2		RS—Factor 3		RS—Factor 4	
Variable	*r*	*p*	*r*	*p*	*r*	*p*	*r*	*p*	*r*	*p*
**SWBQp variables**	0.412 **	<0.001								
Personal	0.485 **	<0.001	0.547 **	<0.001	0.285 **	0.003	0.365 **	<0.001	0.418 **	<0.001
Communal	0.281 **	0.004	0.329 **	<0.001	0.126	0.201	0.225 *	0.022	0.228 *	0.020
Environmental	0.322 **	<0.001	0.444 **	<0.001	0.119	0.229	0.251 *	0.010	0.196 *	0.047
Transcendental	0.293 *	0.003	0.369 **	<0.001	0.074	0.453	0.231 *	0.018	0.243 *	0.013
**FAS variables**	−0.026	0.790								
Centrality of parenting	−0.026	0.790	−0.137	0.165	−0.149	0.132	−0.019	0.849	−0.249 *	0.011
Suspended Life	−0.105	0.287	−0.098	0.324	−0.065	0.506	−0.055	0.582	−0.206 *	0.036
Acceptation of life without children	0.227 *	0.021	0.218 *	0.027	0.214 *	0.029	0.066	0.505	0.290 **	0.003

** Correlation is significant at the 0.01 level (2-tailed); * Correlation is significant at the 0.05 level (2-tailed).

## Data Availability

The data analyzed in the study are available upon request to the corresponding author.
